# Early inflorescence development in the grasses (Poaceae)

**DOI:** 10.3389/fpls.2013.00250

**Published:** 2013-07-23

**Authors:** Elizabeth A. Kellogg, Paulo E. A. S. Camara, Paula J. Rudall, Philip Ladd, Simon T. Malcomber, Clinton J. Whipple, Andrew N. Doust

**Affiliations:** ^1^Department of Biology, University of Missouri-St. LouisSt. Louis, MO, USA; ^2^Department of Botany, University of BrasiliaBrasilia, Brazil; ^3^Jodrell Laboratory, Royal Botanic GardensKew, Richmond, UK; ^4^School of Veterinary and Life Sciences, Murdoch UniversityPerth, WA, Australia; ^5^Department of Biology, California State University-Long BeachLong Beach, CA, USA; ^6^Department of Biology, Brigham Young UniversityProvo, UT, USA; ^7^Department of Botany, Oklahoma State UniversityStillwater, OK, USA

**Keywords:** phyllotaxis, shoot apical meristem, phylogeny, branching, APO1

## Abstract

The shoot apical meristem of grasses produces the primary branches of the inflorescence, controlling inflorescence architecture and hence seed production. Whereas leaves are produced in a distichous pattern, with the primordia separated from each other by an angle of 180°, inflorescence branches are produced in a spiral in most species. The morphology and developmental genetics of the shift in phyllotaxis have been studied extensively in maize and rice. However, in wheat, *Brachypodium*, and oats, all in the grass subfamily Pooideae, the change in phyllotaxis does not occur; primary inflorescence branches are produced distichously. It is unknown whether the distichous inflorescence originated at the base of Pooideae, or whether it appeared several times independently. In this study, we show that *Brachyelytrum*, the genus sister to all other Pooideae has spiral phyllotaxis in the inflorescence, but that in the remaining 3000+ species of Pooideae, the phyllotaxis is two-ranked. These two-ranked inflorescences are not perfectly symmetrical, and have a clear “front” and “back;” this developmental axis has never been described in the literature and it is unclear what establishes its polarity. Strictly distichous inflorescences appear somewhat later in the evolution of the subfamily. Two-ranked inflorescences also appear in a few grass outgroups and sporadically elsewhere in the family, but unlike in Pooideae do not generally correlate with a major radiation of species. After production of branches, the inflorescence meristem may be converted to a spikelet meristem or may simply abort; this developmental decision appears to be independent of the branching pattern.

## Introduction

Inflorescence development controls plant reproduction and hence, fitness. The number of branches produced, and the pattern and timing of their production, dictate the number of flowers, the number of vascular bundles entering the inflorescence (Piao et al., 2009; Zhu et al., 2010), and the way flowers interact with the airstream for pollination (Friedman and Harder, 2004, 2005). In the cereals, in which each flower can produce only one seed, the number of flowers controls the potential number of seeds. In addition, the vascular (hydraulic) architecture of the inflorescence affects the ability of the plant to supply developing seeds with water and photosynthate. Thus, inflorescence architecture controls both the number and the size of seeds. Seed number and size are central demographic parameters in the wild and also critical economic parameters in cereal grain production, where together they determine yield. In other words, the structure of the inflorescence has obvious economic implications in crops and profound ecological implications in wild plants. Because of the importance of inflorescence architecture, much effort has gone in to describing phenotypic and genetic aspects of inflorescence development, but this work has focused on a few model species (e.g., *Arabidopsis thaliana*) and a couple of hugely important crops (rice, *Oryza sativa*, and maize, *Zea mays*). Much less work has been done to extend these data to wild species.

Inflorescence development in the grass family (Poaceae) begins when the shoot apical meristem converts from its vegetative state, producing leaves on its flanks, to an inflorescence meristem. Bracts form as in many other flowering plants, but their growth is suppressed (Evans, 1940; Latting, 1972; Fraser and Kokko, 1993; Chuck et al., 2010; Whipple et al., 2010); the mature inflorescence is thus ebracteate. Neither the inflorescence meristem nor the branch meristems are ever converted directly to floral meristems. Instead all higher-order meristems produced by the inflorescence meristem and its branches are ultimately converted to spikelet meristems, which first produce two bracts known as glumes, followed by one or more flowers in tiny spikes (hence the term spikelet). Because the development of the spikelet is highly stereotyped and deterministic within most major groups of grasses, investigations of inflorescence architecture treat the spikelet as the terminal differentiated unit of the inflorescence, rather than the flower. In short, the inflorescence meristem may produce either branch meristems or spikelet meristems on its flanks, and the branch meristems may themselves produce either branch meristems or spikelet meristems. The inflorescence meristem itself may ultimately be converted to a spikelet meristem, or may simply cease to produce lateral structures; in the latter case, it ends blindly. By viewing meristem fate as a limited set of developmental decisions, it has been possible to produce models of inflorescence development (Kellogg, 2000; Prusinkiewicz et al., 2007).

In grasses, as in many other flowering plants, the phyllotaxis of lateral structures in the inflorescence may continue the same phyllotactic pattern as the leaves, or it may change. In all grasses and their close relatives in the “core” Poales [the clade consisting of Anarthriaceae, Centrolepidaceae, Restionaceae, Flagellariaceae, Joinvilleaceae, Ecdeiocoleaceae, and Poaceae (Michelangeli et al., 2003)], the vegetative meristem produces leaves in a distichous pattern (Stevens, 2012). In some species of grasses (e.g., barley, wheat), the distichous pattern of the vegetative meristem is preserved through the transition to flowering so that the primary branches of the inflorescence are also distichous (Bonnett, 1935, 1936; Moncur, 1981). In rice and maize, however, conversion to an inflorescence meristem correlates with production of branches in spiral phyllotaxis (Bonnett, 1940; Ikeda et al., 2005).

The literature on inflorescence development in grasses hints at a phylogenetic correlation with inflorescence phyllotaxis, but sampling is uneven (Table 1). Although data are available for nine of the 12 subfamilies of grasses plus two outgroups (Ecdeiocoleaceae and Centrolepidaceae), most sampling has focused on the cereal crops (particularly wheat, rice, and maize), the cool season (C_3_) pasture grasses in subfamily Pooideae, and some of the C_4_ grasses in subfamily Panicoideae.

**Table 1:** **Phyllotaxis of primary inflorescence branches and presence of a terminal flower in the grasses; taxa in which the two-ranked inflorescence is distichous are indicated as “two-ranked (d)”**.

**Species**	**Subfamily**	**Tribe**	**Phyllotaxis**	**Terminal spikelet/flower**	**References**
*Anomochloa marantoidea*	Anomochlooideae		uncertain	yes	Judziewicz and Soderstrom, 1989
*Streptochaeta spicata*	Anomochlooideae		spiral	no (?)	Sajo et al., 2008
*Streptochaeta angustifolia*	Anomochlooideae		spiral	no	This paper
*Pharus latifolius; P. lappulaceus*	Pharoideae		spiral	yes (?)	Sajo et al., 2007
*Oryza sativa*	Ehrhartoideae	Oryzeae	spiral	no	Moncur, 1981; Ikeda et al., 2005
*Zizania aquatica*	Ehrhartoideae	Oryzeae	spiral	not determined	Weir and Dale, 1960; Liu et al., 1998
*Brachyelytrum erectum*	Pooideae	Brachyelytreae	spiral	yes	This paper
*Nardus stricta*	Pooideae	Nardeae	two-ranked	yes	This paper
*Phaenosperma globosa*	Pooideae	Phaenospermateae	two-ranked (d)		This paper
*Nassella filiculmis*	Pooideae	Stipeae	two-ranked (d)	yes	This paper
*Nassella manicata*	Pooideae	Stipeae	two-ranked (d)	yes	This paper
*Nassella tenuissima*	Pooideae	Stipeae	two-ranked	yes	This paper
*Melica nitens*	Pooideae	Meliceae	two-ranked	not determined	This paper
*Melica macra*	Pooideae	Meliceae	two-ranked	not determined	This paper
*Glyceria striata*	Pooideae	Meliceae	two-ranked (d)	yes	This paper
*Diarrhena obovata*	Pooideae	Diarrheneae	two-ranked (d)	not determined	This paper
*Brachypodium distachyon*	Pooideae	Brachypodieae	two-ranked (d)	yes	This paper
*Brachypodium retusum*	Pooideae	Brachypodieae	two-ranked (d)	yes	This paper
*Elymus hystrix*	Pooideae	Triticeae	two-ranked (d)	yes	This paper
*Elymus repens*	Pooideae	Triticeae	two-ranked (d)	yes	Evans, 1940; Sharman, 1945
*Hordeum vulgare*	Pooideae	Triticeae	two-ranked (d)	no	Bonnett, 1935; Moncur, 1981; Babb and Muehlbauer, 2003; this paper
*Secale cereale*	Pooideae	Triticeae	two-ranked (d)	no	Moncur, 1981
*Triticum aestivum*	Pooideae	Triticeae	two-ranked (d)	yes	Bonnett, 1936; Moncur, 1981
*Triticum turgidum*	Pooideae	Triticeae	two-ranked (d)	no	Moncur, 1981
*Arrhenatherum elatius*	Pooideae	Poeae	two-ranked (d)	yes	Evans, 1940
*Avena fatua*	Pooideae	Poeae	two-ranked (d)	yes	Landes and Porter, 1990
*Avena sativa*	Pooideae	Poeae	two-ranked (d)	yes	Bonnett, 1937; Moncur, 1981; this paper
*Cynosurus cristatus*	Pooideae	Poeae	two-ranked (d)	not determined	Latting, 1972
*Dactylis glomerata*	Pooideae	Poeae	two-ranked (d)	yes	Evans, 1940; Fraser and Kokko, 1993
*Deschampsia caespitosa*	Pooideae	Poeae	two-ranked (d)	not determined	Latting, 1972
*Lolium perenne*	Pooideae	Poeae	two-ranked (d)	yes	Evans, 1940
*Phalaris canariensis*	Pooideae	Poeae	two-ranked (d)	yes	Evans, 1940
*Phalaris arundinacea*	Pooideae	Poeae	two-ranked (d)	yes	Moncur, 1981
*Phleum pratense*	Pooideae	Poeae	two-ranked (d)	yes	Evans, 1940
*Poa arctica*	Pooideae	Poeae	two-ranked (d)	yes	Latting, 1972
*Poa alpina*	Pooideae	Poeae	two-ranked (d)	not determined	Latting, 1972
*Trisetum spicatum*	Pooideae	Poeae	two-ranked (d)	not determined	Latting, 1972
*Brachiaria decumbens*	Panicoideae	Paniceae	spiral	no	Stür, 1986
*Cenchrus* spp. (including *Pennisetum*)	Panicoideae	Paniceae	spiral	no	Doust and Kellogg, 2002
*Echinochloa frumentacea*	Panicoideae	Paniceae	spiral	yes	Moncur, 1981
*Digitaria phaeothrix*	Panicoideae	Paniceae	spiral	not determined	Rua and Boccaloni, 1996
*Eriochloa* spp.	Panicoideae	Paniceae	spiral	No	Reinheimer et al., 2009
*Ixophorus unisetus*	Panicoideae	Paniceae	spiral	not determined	Kellogg et al., 2004
*Megathyrsus maximus* (=*Panicum maximum*)	Panicoideae	Paniceae	spiral	yes	Reinheimer et al., 2005
*Melinis* spp.	Panicoideae	Paniceae	two-ranked (d)	yes	Reinheimer et al., 2009
*Moorochloa eruciformis*	Panicoideae	Paniceae	two-ranked (d)	yes	Reinheimer et al., 2009
*Panicum miliaceum*	Panicoideae	Paniceae	spiral	yes	Moncur, 1981; Bess et al., 2005
*Setaria* spp.	Panicoideae	Paniceae	spiral	no	Doust and Kellogg, 2002
*Urochloa* spp.	Panicoideae	Paniceae	spiral and two-ranked (d)	variable	Reinheimer et al., 2005, 2009
*Zuloagaea bulbosa*	Panicoideae	Paniceae	spiral	yes	Bess et al., 2005
*Paspalum haumanii*	Panicoideae	Paspaleae	spiral	not determined	Rua and Weberling, 1995
*Bothriochloa bladhii*	Panicoideae	Andropogoneae	spiral	uncertain	LeRoux and Kellogg, 1999
*Coelorachis aurita*	Panicoideae	Andropogoneae	two-ranked	yes	LeRoux and Kellogg, 1999
*Heteropogon contortus*	Panicoideae	Andropogoneae	two-ranked	uncertain	LeRoux and Kellogg, 1999
*Hyparrhenia hirta*	Panicoideae	Andropogoneae	uncertain	yes	LeRoux and Kellogg, 1999
*Sorghum bicolor*	Panicoideae	Andropogoneae	spiral	yes	Moncur, 1981; Brown et al., 2006
*Zea mays*	Panicoideae	Andropogoneae	spiral	no	Bonnett, 1940; Moncur, 1981
*Chionochloa macra*	Danthonioideae		two-ranked (d)	yes	Martin et al., 1993
*Chloris barbata*	Chloridoideae	Cynodonteae	spiral	no	Liu et al., 2007
*Cynodon dactylon*	Chloridoideae	Cynodonteae	spiral	no	Liu et al., 2007
*Dactyloctenium aegypticum*	Chloridoideae	Cynodonteae	spiral	no	Liu et al., 2007
*Eleusine coracana*	Chloridoideae	Cynodonteae	spiral	no	Moncur, 1981
*Eleusine indica*	Chloridoideae	Cynodonteae	spiral	no	Liu et al., 2007
*Microchloa indica*	Chloridoideae	Cynodonteae	uncertain	no	Liu et al., 2007
*Eragrostis tef*	Chloridoideae	Eragrostideae	spiral	yes	Moncur, 1981

In a few genera the inflorescence has a single primary branch; these are listed as having uncertain phyllotaxis.

(?) indicates uncertainty in interpretation.

Of the families in the core Poales, only Ecdeiocoleaceae and Centrolepidaceae have been studied developmentally. In representatives of both families, the inflorescence meristem produces lateral structures in a spiral. The meristem of *Ecdeiocolea monostachya* produces large bracts on its flanks, with floral meristems forming in the axils of the bracts. The inflorescence meristem thus produces floral meristems directly (Rudall et al., 2005). The bracts in *Centrolepis* are less prominent but otherwise the pattern appears to be similar (Sokoloff et al., 2009a,b). The inflorescence meristem of *Ecdeiocolea* appears not to terminate in a flower, but rather produces smaller and smaller bracts that eventually fail to produce a flower in their axils (Ladd, personal observation). The fate of the inflorescence meristem in *Centrolepis* is unknown, but if it ultimately becomes a flower, this must occur late enough in development that it has not been observed in developmental studies. Sokoloff et al. (2009b) note that the primary inflorescence bracts of *C. racemosa* are reduced in size toward the apex of the inflorescence, hinting that the pattern may be similar to that in *Ecdeiocolea*.

The subfamily Anomochlooideae is sister to the remainder of the grasses, and includes the genera *Streptochaeta* and *Anomochloa* [Grass Phylogeny Working Group II, 2012; (GPWG II)], neither of which produces spikelets. The inflorescence meristem of *Streptochaeta* produces primary branches (sometimes called “spikelet equivalents”) that terminate in flowers; while initiation of these branches is not documented, figures of slightly later stages suggest that they are arranged in a spiral (Sajo et al., 2008). The fate of the inflorescence meristem is not described. *Anomochloa*, in contrast, is reported to be primarily distichous (Judziewicz and Soderstrom, 1989), although again definitive data are not available. Sajo et al. (2012) provide a careful description of the development of the primary inflorescence branches, but the arrangement of these branches in relation to the main axis is not reported.

Subfamily Pharoideae, with four genera, is sister to all grasses except Anomochlooideae; like all grasses other than Anomochlooideae, members of Pharoideae produce spikelets. The immediate products of the inflorescence meristem of *Pharus* are branches that appear to be spirally arranged, and the apical meristem terminates in a spikelet (Sajo et al., 2007). Data are unavailable for Puelioideae and Bambusoideae. In subfamily Ehrhartoideae, tribe Oryzeae, the inflorescence meristem produces branches in a spiral pattern in *Oryza sativa* (rice) and *Zizania aquatica* (wildrice) (Moncur, 1981; Liu et al., 1998); the inflorescence meristem itself ultimately aborts. No data are available for members of the other tribes in Ehrhartoideae.

The subfamilies Panicoideae, Aristidoideae, Chloridoideae, Micrairoideae, Arundinoideae, and Danthonioideae (the PACMAD clade) together include about 60% of grass species; within this large clade most data come from subfamily Panicoideae, tribe Paniceae. The inflorescence meristems of most species produce branches in a spiral, forming multiple orthostichies or parastichies depending on the shape of the axis (Table 1). In some cases, the inflorescence meristem ultimately converts to a spikelet meristem and in others it simply terminates without further differentiation. Likewise, in tribe Andropogoneae, *Bothriochloa bladhii, Sorghum bicolor*, and *Zea mays* produce branches in a spiral (Bonnett, 1940; LeRoux and Kellogg, 1999; Brown et al., 2006), but the fate of the inflorescence meristem differs between species. All studied species in subfamily Chloridoideae have spiral phyllotaxis and lack a terminal spikelet (Moncur, 1981; Liu et al., 2007). We have found no published data for Aristidoideae, Micrairoideae, or Arundinoideae, although several arundinoids are reported to have spiral phyllotaxis (J. K. Teisher, Washington University, pers. communication).

Even though spiral phyllotaxis is widespread in the grasses and common in many outgroups, two-ranked phyllotaxis also occurs (Table 1). If the two ranks of primary branches initiate at angles of 180°, we refer to them as distichous; if the ranks are less than 180° apart on one side of the inflorescence we simply use the term two-ranked. Thus, “distichous” is a subset of “two-ranked.” The only data for subfamily Danthonioideae come from *Chionochloa macra*, in which the primary branch primordia are distichous (Martin et al., 1993). Inflorescences with two ranks of branches have also been described for Panicoideae tribes Paniceae [e.g., *Urochloa*, distichous (Reinheimer et al., 2005), and Andropogoneae, not consistently distichous (LeRoux and Kellogg, 1999; Kellogg, 2000)]. Most notably, inflorescences of all studied members of Triticeae and Poeae (subfamily Pooideae) are apparently distichous (Table 1). Based on current data, the shift in inflorescence phyllotaxis from spiral to distichous appears to have occurred at about the same time as the expansion of genome size and shift in chromosome number that characterizes Triticeae, Bromeae, and Poeae (Grass Phylogeny Working Group, 2001; Kellogg and Bennetzen, 2004), although there is no obvious mechanistic reason why genome size *per se* should affect inflorescence architecture. Alternatively, distichous phyllotaxis could be a synapomorphy for Pooideae, and could correlate with the ecological expansion of the group to temperate climates (Grass Phylogeny Working Group, 2001; Edwards and Smith, 2010; Grass Phylogeny Working Group II, 2012). However, establishing this correlation requires data on members of the tribes Brachyelytreae, Nardeae, Stipeae, Phaenospermateae, Meliceae, and Diarrheneae, which are successive sister groups to the rest of the subfamily and have not been studied.

To determine the phylogenetic patterns that will drive investigations of gene evolution, we analyze the developmental fate of the inflorescence meristem in grasses, and consider the phyllotaxis of the primary branch meristems and whether the meristem converts to a spikelet or simply aborts. We present evidence that spiral phyllotaxis may be ancestral in the grasses, and that two-ranked phyllotaxis is a synapomorphy for a large clade within the subfamily Pooideae. We also identify a set of taxa that exhibit a character state that we call biased distichous [following the terminology of Ikeda et al. (2005)] and we find that even in inflorescences that are initially distichous, the inflorescence is one-sided, developing a clear front and back. While often obvious in figures, this pattern has not been noted by previous authors. Based on our data plus the grass phylogeny, we show that two-ranked inflorescences have arisen independently in the Pooideae and Panicoideae, and that “panicles” (i.e., branched inflorescences) in the two subfamilies develop from different starting points. Formation of a terminal spikelet varies independently of inflorescence phyllotaxis. We conclude with some hypotheses of the possible genetic controls of this development.

## Materials and methods

Inflorescences were collected at appropriate developmental stages and fixed in formalin:acetic acid:ethanol (FAA). The material was then transferred to 70% ethanol for storage. In a few cases, fixed material was rehydrated and infused with osmium tetroxide (OsO_4_) at this stage, using the OTOTO or OTO method of Murphy (1978), as applied by Doust and Kellogg (2002). Inflorescences, whether treated with osmium tetroxide or not, were dehydrated in an ethanol series (70, 80, 90, 95, 100, 100%), dried in a critical point dryer, sputter coated, and examined using a scanning electron microscope. Vouchers of representative specimens are listed in Table 2. The SEM data were collected over a period of years on a variety of machines including an Amray 1000 (at Harvard University, Cambridge, MA, USA), Philips XL20 (at Murdoch University, Perth, WA), Hitachi cold field emission SEM S-4700-II (at the Royal Botanic Gardens, Kew, UK), Hitachi S450 (at the University of Missouri-St. Louis, St. Louis, MO), and Hitachi S-2600H (at Washington University, St. Louis, MO, USA). Images were either captured on Polaroid film (Harvard, UM-St. Louis) and then scanned, or captured digitally (all other sources). Tonal values, brightness, and contrast were adjusted in Adobe Photoshop; in five images embedded scale bars were digitally removed. Images were otherwise unaltered from the originals.

**Table 2:** **Voucher specimens**.

**Species**	**Collector number (herbarium)**	**Locality**
*Aphelia brizula*	Kellogg & Ladd 1015 (MO)	Sullivan's Rock, near Perth, Western Australia
*Centrolepis aristata*	Kellogg & Ladd 1016 (MO)	Sullivan's Rock, near Perth, Western Australia
*Joinvillea ascendens*	NTBG 800379	National Tropical Botanical Garden
*Streptochaeta angustifolia*	Malcomber 3123 (MO)	Seeds originally from Lynn Clark, Iowa State University
*Brachyelytrum erectum*	Kellogg 466 (MO)	Shaw Nature Reserve, MO
*Nardus stricta*	1955-20910	RBG Kew, living accession
*Phaenosperma globosa*	1997-6146	RBG Kew, living accession
*Nassella filiculmis*	1978-1236	RBG Kew, living accession (as *Stipa filiculmis*)
*Nassella manicata*	1987-1267	RBG Kew, living accession (as *Stipa formicarum*)
*Nassella tenuissima*	1978-1226	RBG Kew, living accession (as *Stipa tenuissima*)
*Melica nitens*	Woodbury 1 (MO)	Shaw Nature Reserve, MO (representative of population)
*Melica macra*	1974-469	RBG Kew, living accession
*Glyceria striata*	Kellogg 486 (MO)	Shaw Nature Reserve, MO
*Diarrhena obovata*	Kellogg 468 (MO)	Shaw Nature Reserve, MO
*Brachypodium distachyon*	No voucher	Material from USDA Plant Introduction System
*Brachypodium retusum*	1981-527	RBG Kew, living accession
*Elymus hystrix*	Kellogg 1161 (UMSL)	Shaw Nature Reserve, MO
*Avena sativa* “Albion”	CIav 1012 (USDA-ARS)	Seed from National Small Grains Collection
*Hordeum vulgare* “Abyssinicum”	No voucher	Material from USDA Plant Introduction System

Species were scored for two inflorescence characters, phyllotaxis and presence of a terminal spikelet, and a data matrix was assembled in Mesquite (Maddison and Maddison, 2002–2009). The phylogeny follows that of the Grass Phylogeny Working Group II (Grass Phylogeny Working Group II, 2012), pruned to include taxa for which data are available, plus placeholders for major clades for which data are missing. Thus, for example, we represented Paniceae with all species with published data, but Puelioideae and Bambusoideae were included even though no data are available. Taxa not in the GPWG II phylogeny were placed according to Givnish et al. (2010, outgroups), Quintanar et al. (2007, Aveneae), and Salariato et al. (2010, Melinidinae). Data were mapped on the phylogeny using parsimony ancestral states. To estimate branch lengths for maximum likelihood mapping, the GPWG II phylogeny was pruned to include only those taxa for which we had inflorescence data and a maximum likelihood phylogeny was constructed in MEGA 5.0 (Tamura et al., 2011). The tree was opened in Mesquite (Maddison and Maddison, 2002–2009), and the characters mapped using maximum likelihood.

In our descriptions we use the term “two-ranked” to mean any inflorescence in which the primary branches form two orthostichies. When the two ranks are separated by an angle of approximately 180°, we refer to the phyllotaxis as “distichous.” Inflorescences with more than two ranks are called spiral, polystichous, or having multiple orthostichies. However, as noted below many of these “spiral” inflorescences actually do not have identical angles between successive branches, and thus do not conform to a standard spiral based on Fibonacci or Lucas numbers.

## Results

### Outgroup taxa

#### *Aphelia brizula* (Centrolepidaceae)

**Figure 1:**
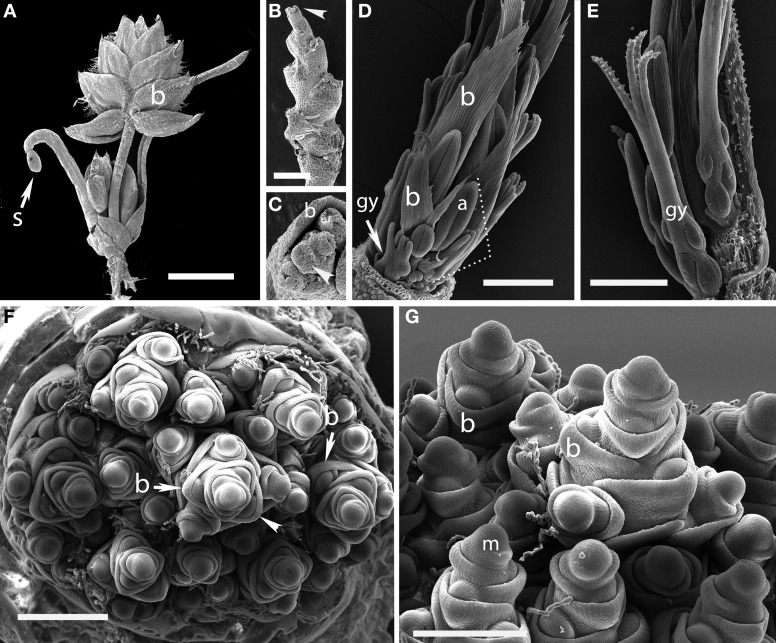
**Outgroups. (A–C)** *Aphelia brizula*, showing distichous phyllotaxis. **(A)** entire plant; **(B)** inflorescence with bracts removed; **(C)** close-up of the apex of **(B)**; arrow indicates one of four visible gynoecia. The apex terminates in a set of increasingly small gynoecia that appear to lack bracts. **(D,E)** *Centrolepis aristata*, showing spiral phyllotaxis. **(D)** Dotted lines indicate one floral unit. (**F,G)** *Joinvillea ascendens*, showing spiral phyllotaxis; it is unclear at this stage of development whether the meristems will develop into branches or spikelets. b, bract; gy, gynoecium; a, anther; m, meristem; s, seed coat. Arrow head in **(B)** indicates region enlarged in **(C)**. Scale bars: **(A)**, 3 mm; **(B)**, 1 mm; **(D–F)**, 500 μm; **(G)**, 400 μm.

Plants of this species are tiny enough to fit entirely on an SEM stub (Figures 1A,B). The inflorescence bears striking large, distichous bracts, each with a fimbriate edge; when these are removed the underlying distichy of the shoot is clear. Although the entire inflorescence looks superficially like a grass spikelet, each bract subtends one or more naked flowers. The more proximal bracts subtend staminate flowers, whereas the distal bracts subtend pistillate flowers, each consisting of a single gynoecium. The bracts appear to be smaller closer to the apical meristem, and ultimately cease to be produced altogether, such that the uppermost bract surrounds a set of several gynoecia, which themselves appear less well-developed distally (Figure 1C).

#### *Centrolepis aristata* (Centrolepidaceae)

The floral units of this species are clearly arranged in a spiral (Figures 1D,E). Each floral unit (variously interpreted as a flower or pseudanthium) consists of a multicarpellate gynoecium plus a single stamen and a bract [called a “bract-like phyllome” by Sokoloff et al. (2009a)]. We did not observe the apical meristem.

#### *Joinvillea ascendens* (Joinvilleaceae)

The inflorescence of *Joinvillea* is large, up to 40 cm long, and multibranched. Each branch and each flower is subtended by a small bract, as also shown by Whipple et al. (2007), Preston et al. (2009), and Sajo and Rudall (2012). Because of difficulty of acquiring appropriate material, we have only limited data. The earliest stage we have observed shows a large number of presumed branch primordia arising from a broad meristem (Figure 1F). While this stage is too late to be certain of the phyllotaxis of the primary branches it appears consistent with a spiral arrangement. Each individual branch primordium itself has spiral phyllotaxis, with prominent bracts (Figures 1F,G). Tentatively, then we describe the inflorescence of *Joinvillea* as spiral.

### Poaceae subfamily anomochlooideae

#### Streptochaeta angustifolia

**Figure 2:**
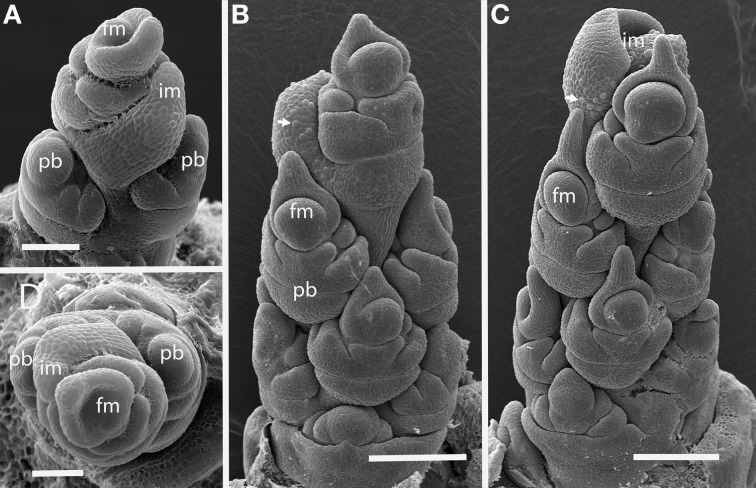
***Streptochaeta angustifolia* (Anomochlooideae).** The first two primary branch primordia (pb) form at an angle less than 180° **(A,D)**; subsequent branches are initiated in a spiral **(B,C)**. The inflorescence meristem appears to terminate in a set of small bracts, identifiable by their broad shape and obvious trichomes (arrows). Fm, floral meristem; im, inflorescence meristem; pb, primary branch; Scale bars: **(A),** 100 μm; **(B, C),** 200 μm; **(D),** 100 μm.

The first two branches of the inflorescence are separated by an angle of 105° (Figures 2A,D), with subsequent branching establishing spiral phyllotaxis. Each floral unit of *Streptochaeta* is subtended by a broad structure that appears to be a reduced bract; this forms trichomes early in development that initially appear as round bumps (Figures 2B,C). As the inflorescence matures, the apex produces bracts that appear to lack floral structures (Figures 2C,D).

### Poaceae subfamily pooideae

#### Tribe brachyelytreae

**Figure 3:**
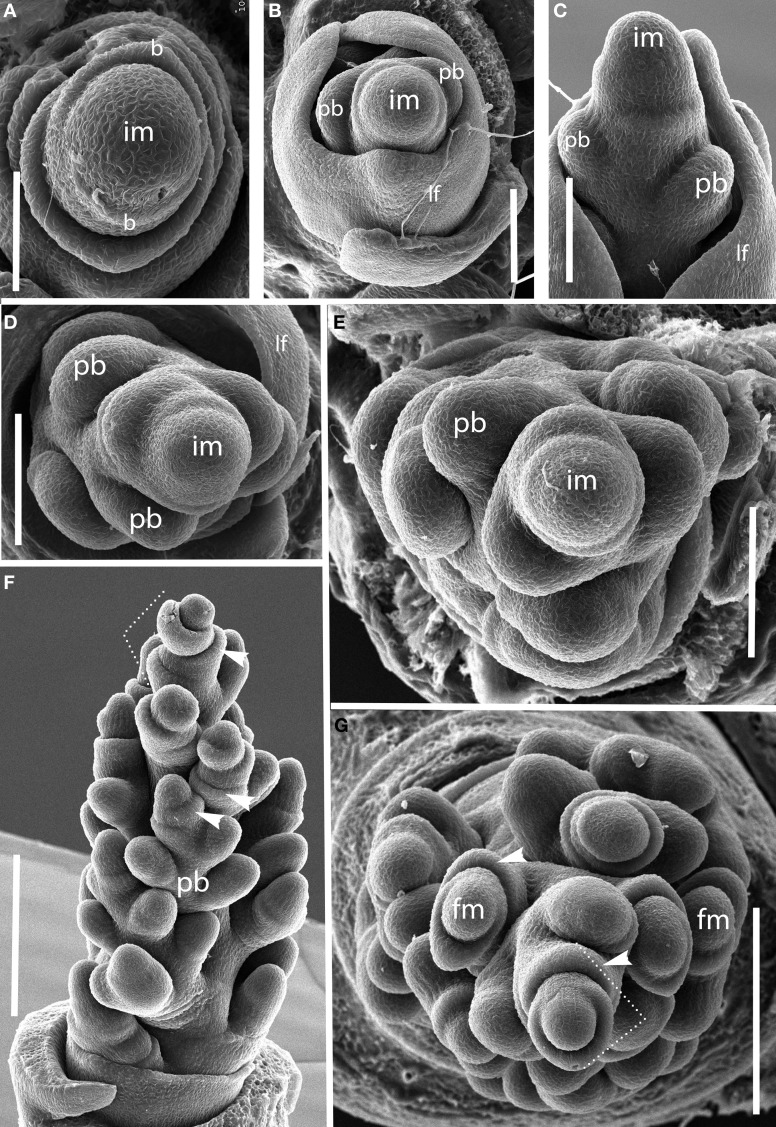
***Brachyelytrum erectum* (Brachyelytreae). (A)** Inflorescence meristem just after transition to flowering. **(B–E)** Successively later stages of development, showing multiple orthostichies; note that branches on one side are displaced by 180°. **(F,G).** Initiation of glumes and lemmas, showing terminal spikelet (dotted lines). b, bract; fm, floral meristem; im, inflorescence meristem; lf, leaf; pb, primary branch; arrows, glumes. Scale bars: **(A–E)**, 100 μm; **(F)**, 250 μm; **(G)**, 200 μm.

***Brachyelytrum erectum (Figure 3).*** The inflorescence of *Brachyelytrum* is multiranked, but it is not a standard spiral. The first two or three branches are separated by an angle of ~180°, and so appear distichous (Figures 3A–C), although the plane of distichy is perpendicular to that of the leaves (Figures 3B,C). Subsequent branches are separated by angles of 120–160° (Figures 3D,E). The result is a multi-ranked inflorescence with a distinct “front” (bearing branches) and “back” (with no branches) (Figure 3E). As the branches themselves branch, the unbranched side is obscured by higher order branches (Figures 3F,G). In the inflorescence shown, the unbranched side is the side away from the mid-rib of the uppermost leaf, but this orientation was not universally observed. The inflorescence is terminal on the stem, so the front-back structure is not obviously related to any existing axis. The axis ultimately ends in a one-flowered spikelet (Figures 3F,G).

#### Tribe nardeae

**Figure 4:**
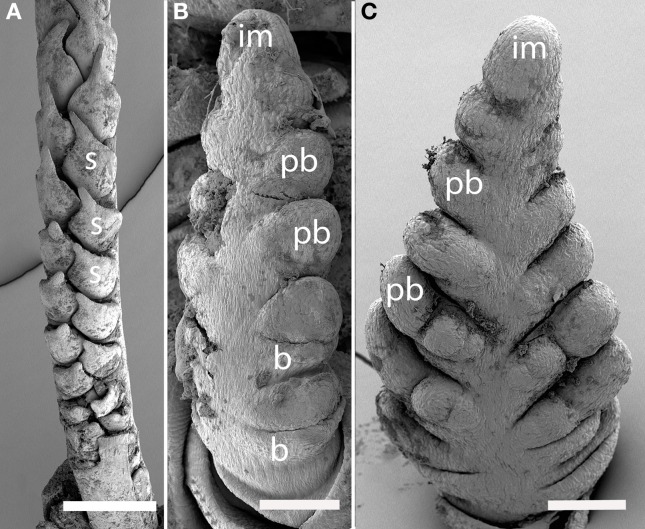
***Nardus* (Nardeae) and *Phaenosperma* (Phaenospermateae). (A)** *Nardus stricta*. Spikelets are two-ranked and unilateral. **(B,C)** *Phaenosperma globosa*. Primary branches are two-ranked and approximately distichous **(C)**, secondary branches form on the side facing the camera. s, spikelet; im, inflorescence meristem, pb, primary branch, b, bract, Scale bars: **(A)**, 500 μm; **(B,C)**, 100 μm.

***Nardus stricta (Figure 4A).*** The inflorescence meristem of *Nardus stricta* produces spikelets directly, rather than producing branch meristems. It is thus similar to the lateral branches in many other taxa in which the two ranks of spikelets are both formed on one side of the inflorescence axis, and are separated by an angle appreciably less than 180°. The inflorescence terminates in a spikelet (not shown).

#### Tribe phaenospermateae

***Phaenosperma globosa (Figures 4B,C).*** The inflorescence of *P. globosa* is two-ranked and approximately distichous initially. Higher order branching occurs predominantly on one side. We do not have data on late development to determine the fate of the inflorescence meristem.

#### Tribe stipeae

**Figure 5:**
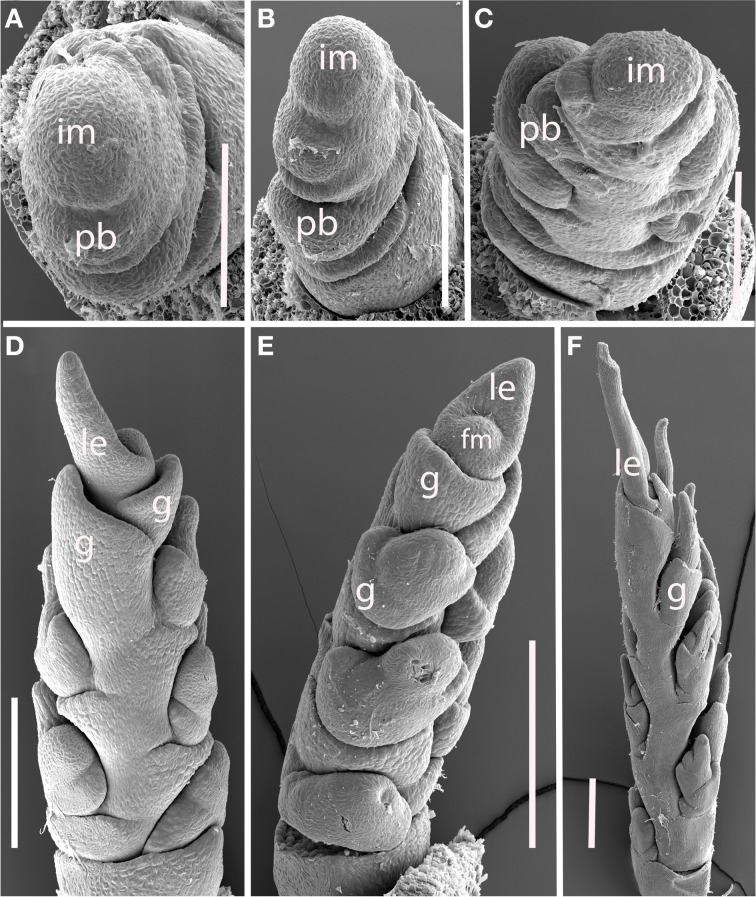
***Nassella filiculmis* (Stipeae). (A,B)** Early development, showing two-ranked nearly distichous branching. **(C)** Branch meristems enlarging on one side of the inflorescence. **(D–F)** Inflorescence becomes unilateral and terminal spikelet differentiates. im, inflorescence meristem; pb, primary branch; fm, floral meristem; g, glume; le, lemma. Scale bars: **(A–C)**, 100 μm; **(D–F)**, 200 μm.

***Nassella spp. (Figure 5).*** Data are presented for *Nassella filiculmis*, but observations for *N. manicata* (= *Stipa formicarum*) and *N. tenuissima* were similar, and we infer that the results are general for the genus. The primary branches of the *Nassella* inflorescence are two-ranked and apparently distichous, separated by a branch angle very close to 180° (Figures 5A,B). However, as the primary branches enlarge and branch again to form secondary branches, additional branching occurs on only one side. The inflorescence develops a clear “back” with no branches and “front” with branches (Figures 5C–F). As with *Brachyelytrum*, the orientation of the branched and unbranched sides does not correlate with any obvious other landmark. The inflorescence meristem terminates in a spikelet and a basipetal pattern of spikelet maturation is established (Figures 5E,F).

#### Tribe meliceae

**Figure 6:**
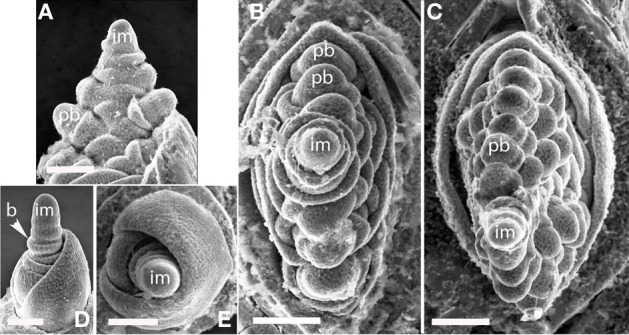
**(A–C),** *Glyceria striata*. **(A,B)** Distichous primary branch formation in the plane of the leaves; **(C)** secondary branches forming on one side of the inflorescence (to the right in the photo). **(D,E)** *Melica nitens* (Meliceae), distichous bract formation. im, inflorescence meristem; pb, primary branch; b, bract. Scale bars: **(A–E)**, 100 μm.

***Glyceria striata.*** The phyllotaxis of the primary branches of *Glyceria* is distichous, with branching occurring in the same plane as the leaves (Figures 6A–C). Higher-order branches, however, form on only one side (Figure 6C).

***Melica spp.*** Data are presented for *Melica nitens*, but observations on *M. macra* are similar, suggesting that the patterns observed are general for the entire genus. Primary branches in *Melica* are clearly distichous (Figures 6D,E). Material at later stages of development was unavailable.

#### Tribe diarrheneae

**Figure 7:**
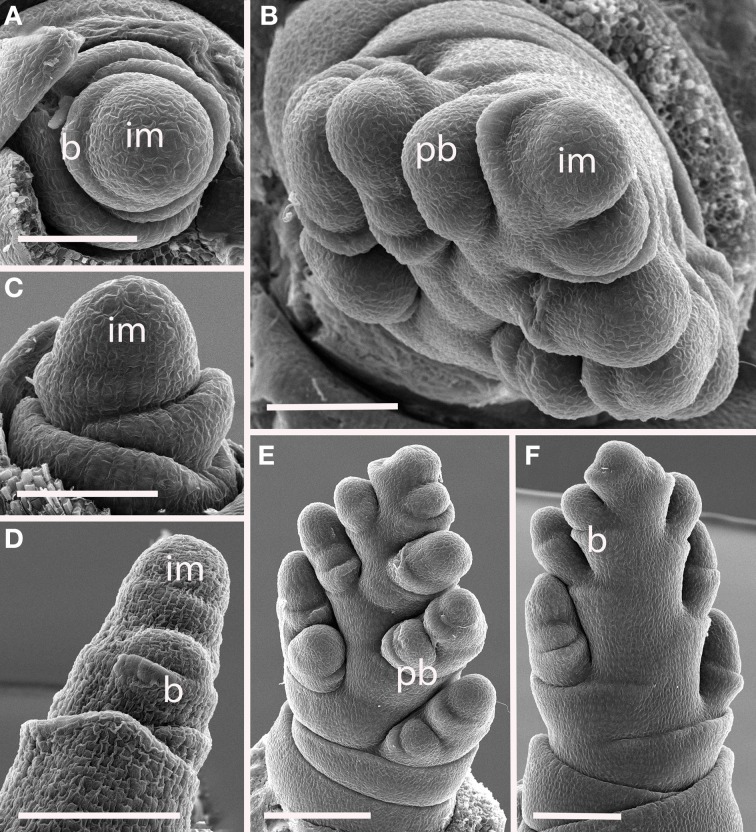
***Diarrhena obovata* (Diarrheneae). (A,C)** Inflorescence meristem shortly after the transition to flowering; **(D)** initiation of a primary branch in the axil of a bract; **(B,E,F)** later development showing formation in primary branches in two, non-distichous, ranks and secondary branches on one side. im, inflorescence meristem; pb, primary branch; b, bract. Scale bars: **(A–D)**, 100 μm; **(E)**, 200 μm; **(F)**, 100 μm.

***Diarrhena obovata (Tribe Diarrheneae) (Figure 7).*** Although the inflorescence meristem produces lateral structures in a distichous pattern in very early development (Figures 7A,C), as the primary branches develop they are separated by an angle of approximately 125° (Figures 7B,D), producing a slightly one-sided, or biased distichous, inflorescence. As with many other inflorescences described here, secondary branches form only on one side of the axis (Figures 7B,E,F). The inflorescence ultimately terminates in a spikelet (Figure 7F).

#### Tribe brachypodieae

**Figure 8:**
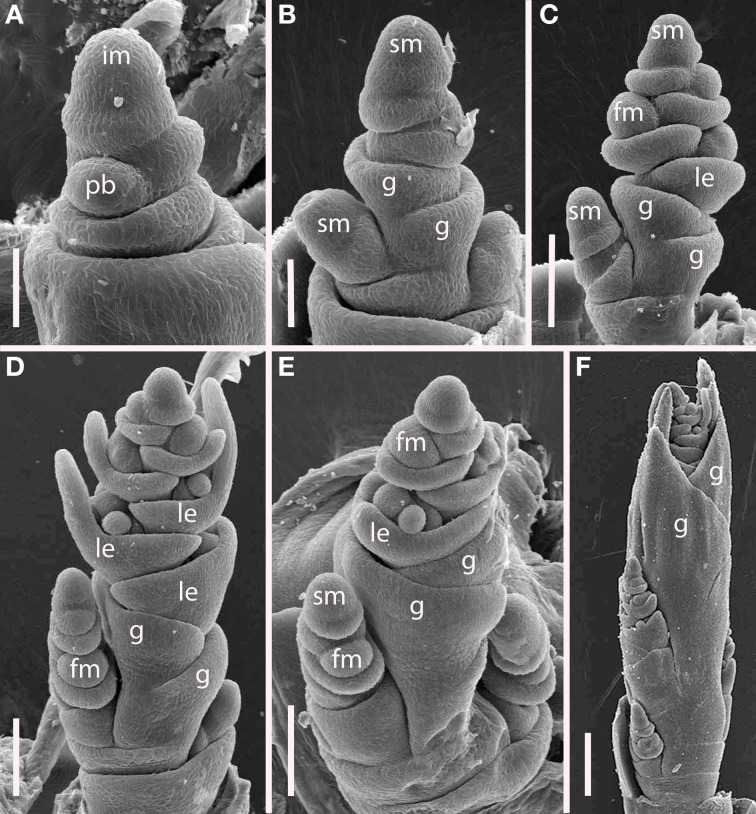
***Brachypodium distachyon* (Brachypodieae). (A)** Distichous primary branch formation; **(B)** inflorescence meristem and uppermost branch meristem converted to spikelet meristems; **(C–F)**, successive stages of development, showing differentiation of the terminal spikelet well ahead of the lateral spikelets. im, inflorescence meristem; pb, primary branch; sm, spikelet meristem, g, glume, le, lemma, fm, floral meristem. Scale bars: **(A,B)**, 50 μm; **(C–E)**, 100 μm; **(F)**, 200 μm.

***Brachypodium distachyon (Figure 8).*** The inflorescences of *B. distachyon* are clearly distichous (Figures 8A,B), a pattern that is also observed in *B. retusum* (not shown). The terminal spikelet differentiates rapidly, well before the few primary branches (Figures 8C–F). All primary branches and the inflorescence axis itself terminate in spikelets.

#### Tribe triticeae

**Figure 9:**
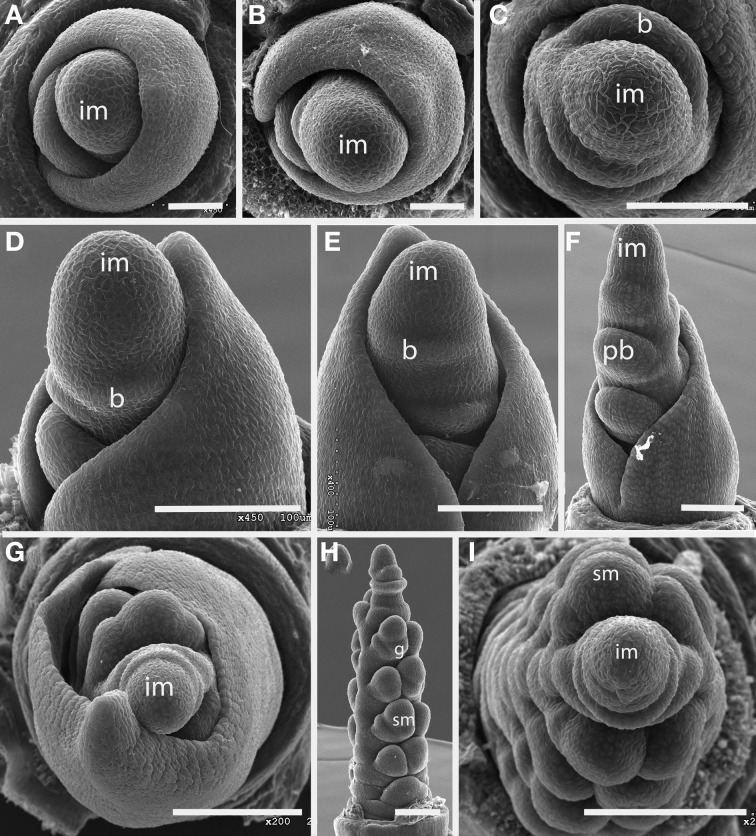
***Elymus hystrix* (Triticeae).** (**A–E**) Successive stages of distichous bract formation in the plane of the leaves; **(F)** initiation of primary branch meristems; **(G–I)**, differentiation of spikelet meristems. im, inflorescence meristem; pb, primary branch; b, bract, sm, spikelet meristem, g, glume. Scale bars: **(A,B**), 50 μm; (**C–F**), 100 μm; (**G–I**), 200 μm.

**Figure 10:**
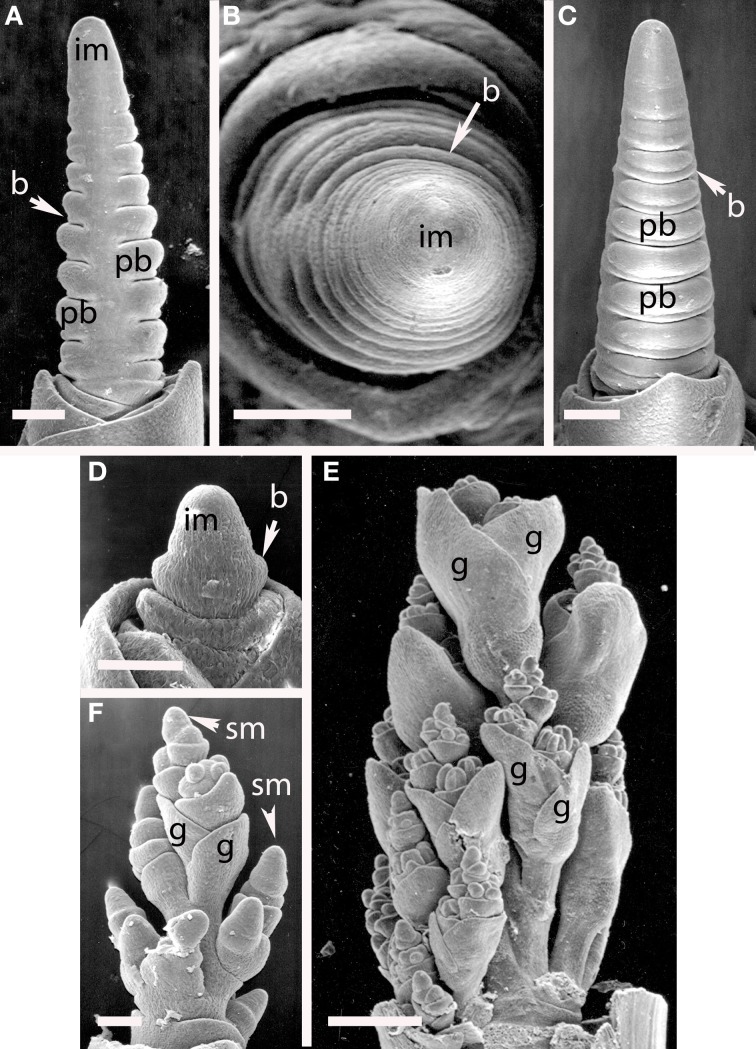
**(A–C)** *Hordeum vulgare* (Triticeae). **(A,C)** Distichous bracts with broad primary branch primordia in their axils; **(B)** distichous bracts. **(D–F)** *Avena sativa* (Poeae). **(D)** distichous bract formation shortly after the transition to flowering; **(E)**, early development of the terminal spikelet; **(F)**, later stage of spikelet development, with additional branching obscuring the primarily distichous pattern. im, inflorescence meristem; pb, primary branch; b, bract; sm, spikelet meristem; g, glume. Scale bars: **(A–E)**, 100 μm, **(F)**, 500 μm.

***Elymus hystrix (Figure 9).*** The inflorescence meristem of *Elymus hystrix* initiates broad bracts in a distichous phyllotaxis; these form in the same plane as the leaves (Figures 10A–E). Branch meristems form in the axils of these bracts (Figure 9F). Each primary branch primordium produces two spikelets, one of which develops ahead of the other (Figures 9G–I). It is tempting to interpret the slower-developing spikelet as the product of a secondary branch, but we do not have evidence to support this interpretation. The inflorescence ultimately terminates in a spikelet (not shown).

***Hordeum vulgare (Figures 10A–C)***. Initiation of primary branches is clearly distichous. Initiation of the branches is preceded by formation of large bracts that remain visible even into early development subtending the broad primary branch meristems. The primary branch meristems will go on to produce three spikelets; the central spikelet is interpreted as being terminal on the primary branch and the two lateral spikelets then represent higher order branches.

#### Tribe poeae

***Avena sativa (Figures 10D–F)***. *Avena sativa* is included here as typical of the Tribe Poeae. Primary branches are distichous and rapidly differentiate into spikelets. The inflorescence meristem converts into a spikelet meristem relatively early in development. Higher order branches form from the primary ones.

#### Origins of two-ranked phyllotaxis and terminal spikelets

**Figure 11:**
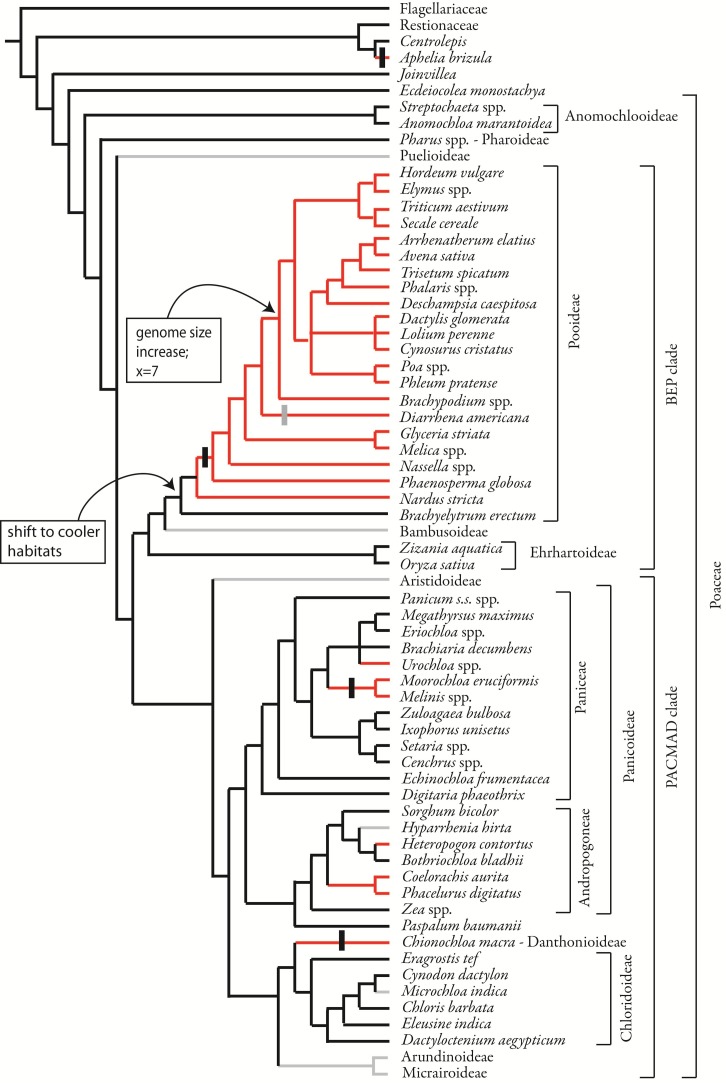
**Phylogenetic distribution of inflorescence phyllotaxis, including data from this study and from the literature.** Parsimony optimization is shown but ML optimization is similar. Species relationships based on Quintanar et al. (2007), Saarela et al. (2010), Salariato et al. (2010), and Grass Phylogeny Working Group II, 2012. Vertical black bars indicate the origin of distichous phyllotaxis, a sub-type of two-ranked; vertical gray bar indicates reversion to non-distichous two-ranked phyllotaxis.

Mapping phyllotaxis on to the phylogeny of grasses shows that the two-ranked inflorescence is synapomorphic for the Pooideae excluding *Brachyelytrum* (Figure 11). Two-ranked inflorescences are derived independently in Centrolepidaceae, and in some members of the PACMAD clade. This result is obtained whether using a parsimony or maximum likelihood optimization of character evolution. While the parsimony optimization places the origin of two-ranked phyllotaxis after the common ancestor of the Pooideae (Figure 11), ML indicates that the marginal probability of the common ancestor having spiral phyllotaxis is 0.73 (not shown); in other words, there is a small probability that two-ranked phyllotaxis originated in the common ancestor and then reversed in *Brachyelytrum*. In addition, there is a small probability (0.27) that the common ancestor of *Nardus* plus the remainder of Pooideae had spiral phyllotaxis; under this model, two-ranked phyllotaxis originated after the divergence of Nardeae. All other origins appear the same with ML and parsimony optimization.

Within the Pooideae with two-ranked inflorescence branching, distichy appears after the divergence of *Nardus*, but is lost in *Diarrhena*. However, in all Pooideae outside Triticeae and Poeae, higher order branching occurs only on one side of the inflorescence, suggesting an underlying bias to the inflorescence that creates a “front” and a “back.”

The inflorescence meristem of most grasses terminates in a spikelet (Table 1). However, blind termination of the axis is common and occurs sporadically in the family. Parsimony optimization of the character indicates that the ancestral state is to produce a terminal spikelet, but within some clades (e.g., Triticeae) the evolution of this character is labile and optimizations are ambiguous (not shown). ML optimizations, in contrast, indicate considerable ambiguity throughout the tree, reflecting the distribution of missing data as well as variation within some clades.

## Discussion

### Morphology

#### Phyllotaxis

Our data show that two-ranked phyllotaxis in Pooideae most likely originated after the common ancestor of the subfamily but before the divergence of Nardeae. This character state thus does not correlate with either the shift to cool habitats or the expansion in genome size. Instead, it originated after the former and well before the latter. In most pooids, the angle between successive primary branches is indeed 180° and the inflorescences appear to follow the phyllotaxis established by the leaves. However, in *Nardus* and *Diarrhena* the inflorescence is not strictly distichous, but is biased to one side, a pattern called “biased distichous” by Ikeda et al. (2005). In some cases this one-sidedness persists to maturity, whereas in others extensive branching and pedicel growth obscure the original developmental pattern.

Our data reinforce the hypothesis that inflorescences with spiral phyllotaxis are likely ancestral in the grasses (Figure 11). However, we also show that this aspect of inflorescence development varies even between closely related genera. In Centrolepidaceae, the inflorescence meristem of *Centrolepis* produces bracts and floral primordia in a spiral, confirming observations of Sokoloff et al. (2009a). *Aphelia*, however, is clearly distichous; this appears to be a derived state. While we infer that the *Joinvillea* inflorescence produces lateral structures in a spiral, we lack data from the very earliest stages to confirm this definitively. However, our data show clearly that the branch meristems in *Joinvillea* are themselves polystichous. We know of no cases in which a distichous inflorescence axis produces primary branches that are spiral, so it seems reasonable to infer that the main axis in *Joinvillea* is itself spiral. Although the first two branches in *Streptochaeta angustifolia* appear to be nearly on opposite sides of the rachis, subsequent branching is clearly spiral, consistent with observations of later stages in *S. spicata* (Judziewicz and Soderstrom, 1989; Sajo et al., 2008).

Whatever the original phyllotaxis, it is common for many inflorescences to have a clear “front” and “back.” It is not clear what determines this apparent front to back axis; we observed no consistent orientation relative to the leaves. In the inflorescences in which the primary branches are apparently distichous, secondary branches form preferentially on one side of the inflorescence (for example, *Nassella, Glyceria*). Even in the spiral inflorescences of *Brachyelytrum erectum*, branches on one side are separated by an angle of almost 180°, whereas angles on the other side are appreciably lower. This one-sided pattern is consistent enough that it appears to represent some sort of biophysical constraint or a regulated, genetically established developmental mechanism. Cresswell et al. (2010) have shown that most pollen is received on a condensed inflorescence on the windward side so this one-sidedness may improve pollen reception. It might also affect the hydraulic architecture of the inflorescence and thus be related to distribution of photosynthate to seeds. Alternatively, it may be a pleiotropic effect of selection on another aspect of inflorescence structure.

The change from distichous vegetative to spiral floral phyllotaxis is often accompanied by a change in the aspect ratio (height:width) of the inflorescence apex. In *Oryza sativa*, the vegetative apex has an aspect ratio of about 1.0, and this drops to 0.4 or even 0.2 in the transition to flowering (Takeoka et al., 1989). In other words, the apex becomes relatively shorter and broader. In the panicoid grasses, the change is in the other direction, toward a taller and narrower inflorescence apex as the transition to flowering is completed (Bonnett, 1940; Reinheimer et al., 2005). In the pooids, the aspect ratio is often much longer than broad (e.g., Figures 6D, 9D), and the phyllotaxis is consistently distichous (Latting, 1972). However, this pattern is not consistent, in that some species with two-ranked phyllotaxis have relatively short broad apices (e.g., Figure 7C); it is possible that these short broad apices correlate with deviations from strict distichy but our sample is not broad enough to test this. Because we investigated a relatively small number of plants for each species, and because accurate measurements are difficult to obtain from SEM photos in which the angle of the specimen is not always perfectly upright, we cannot address the issue of aspect ratio directly with our data. In addition, it is not clear that the aspect ratio would be particularly informative by itself. Phyllotaxis is a function of both the size of the meristem and the size of the primordia it produces, as shown theoretically by Jean (1994) and empirically by Doust (2001). Thus, measuring the size of the meristem is only useful if accompanied by measurements of the size of the primary branch primordia. Nonetheless, our data hint that meristems in Pooideae may be somewhat smaller than those in taxa with spiral phyllotaxis. Phyllotaxis is also affected by the length of time between formation of each successive primordium (the length of the plastochron), which also cannot be determined from our data (Jean, 1994; Doust, 2001).

The relevance of our data to grasses with leafy inflorescences is not clear. Most bamboos and some Andropogoneae have complex flowering shoots that are extensively branched and bracteate, as though the entire structure is neither fully vegetative nor fully floral. Bracts are generally suppressed below the spikelets and below certain inflorescence branches; however, other branches in the inflorescence are subtended by bracts and bear prophylls, hinting at a different pattern of regulation entirely. While it is common for the terminal inflorescence branches in these groups to be two-ranked (but generally not distichous), it is not universal; thus the correlation between inflorescence branching and bract or spathe development remains unclear.

Most descriptions of inflorescence morphology in the grasses attempt to force the variation into a few standard terms borrowed from dicots. Thus, inflorescences in which the spikelets are sessile on the inflorescence axis are called “spikes,” those in which the spikelets are pedicellate are “racemes,” and any inflorescence in which there are higher orders of branching is called a “panicle.” Under this set of definitions, most grasses have some sort of panicle. However, many authors have noted the problems with this approach. One problem is simply that the spikelet itself is a part of an inflorescence and thus is not strictly equivalent to a flower. Because of this, Endress (2010) describes grass inflorescences as “compound spikes.” Other authors use the term co-florescence for all spikelets except the one at the terminus of the inflorescence, which is called the florescence (Vegetti, 1991; Hernández and Rua, 1992; Weberling et al., 1993; Vegetti and Weberling, 1996).

A second problem with using the term “panicle” in the grasses is the sheer diversity of forms. These are diagrammed by Vegetti and Anton (2000). While racemose branching patters are inferred to be ancestral in the monocots, the “panicle” is presumed to be derived (Remizowa et al., 2013). Endress distinguishes “panicles” from either racemose or cymose inflorescences by including those in which the number of orders of branching and the number of flowers (spikelets) produced at any one order are not limited. This definition includes many disparate patterns of development in the grasses.

The data presented here add another dimension to the architectural complexity already documented. “Panicles” in Pooideae originate from primary branches that initiate in a distichous fashion, whereas those in Ehrhartoideae and Panicoideae originate from primary branches initiated in a spiral. Thus, the primary phyllotaxis of the inflorescence is different, even though subsequent branching appears to be morphologically similar.

Phyllotaxis of the inflorescence meristem cannot be determined in all cases, because the term has meaning only if the meristem produces at least two lateral structures. Therefore, in taxa in which there is only one primary branch (e.g., *Hyparrhenia*), the condition is uncertain. In taxa in which the inflorescence meristem produces a single branch and then aborts (e.g., *Microchloa*), the branch then develops in its normal two-ranked pattern. It is easy to imagine that if the inflorescence meristem aborts early, the branch meristem would be wrongly interpreted as the inflorescence meristem. This may be particularly common in the PACMAD clade.

Within the grasses, even if the inflorescence meristem produces primordia in multiple orthostichies or parastichies, higher order meristems (primary, secondary, tertiary branches) always produce two ranks of branch or spikelet primordia; these may or may not be separated by angles of 180°. In addition, the inflorescence meristem itself often shifts from producing branch primordia to producing spikelet primordia; in this case, the spikelet primordia are produced in two ranks. [The lone exception is the staminate inflorescence (tassel) of the domesticated *Zea mays* ssp. *mays*, in which the polystichous phyllotaxis of the long branch meristems is continued through the main axis of the inflorescence; this pattern may simply be a result of domestication selecting for a larger inflorescence meristem (Sundberg et al., 2008)] Thus, the terminal portion of the inflorescence meristem acquires the phyllotaxis of a branch.

#### Termination of the inflorescence

Phyllotaxis of the primary branches is apparently independent of the fate of the inflorescence meristem (Table 1). The inflorescence meristem may be converted to a spikelet, which may develop precociously as shown here for *Brachypodium* and *Nassella*. Alternatively, the meristem may continue producing lateral structures but these may become smaller and smaller, as though the number of meristematic cells becomes increasingly limited; this appears to be the case in several of the grass outgroups but is less obvious in the grasses themselves. More commonly in the grasses, the inflorescence meristem simply terminates blindly, as described for *Oryza*.

The presence or absence of a terminal spikelet is variable between tribes and genera of grasses. Although the majority of species in the family exhibit terminal spikelets, virtually all major clades have several members in which the terminal spikelet is lacking (Butzin, 1979). Reinheimer et al. (2013), in their study of panicoid grasses, show that this character is consistent within some major clades but variable in others. Our data on Pooideae indicates considerable variation. However, because this character is so labile in evolutionary time, a full exploration of its evolution would require more focused sampling than presented here.

### Functional significance

The functional significance of primary branch phyllotaxis is unknown. However, the fact that it can be stable among groups of species, including the several thousand species of subfamily Pooideae, suggests that it is preserved either by selection or developmental constraint. Because inflorescence architecture controls the timing and position of pollen presentation, the timing of seed maturation, the extent of seed provisioning, and the extent of seed dormancy, it is likely to be under selection (Simpson, 1990; González-Rabanal et al., 1994; Friedman and Harder, 2005; Wang et al., 2010; Harder and Prusinkiewicz, 2013). Seed set in grasses appears not to be limited by pollen availability when grasses are growing in dense stands, but may be limiting when plants are widely spread (McKone et al., 1998; Davis et al., 2004); it is thus unclear whether or not pollination efficiency provides a strong selective force. The number of primary branches also correlates with the number of vascular bundles in the peduncle, suggesting that the number may be limited by carbohydrate supply (Terao et al., 2009). Indeed the complexity of the inflorescence suggests that the grasses may be uniquely placed to adjust their seed production in response to a variety of environmental parameters. Nonetheless, the absence of an obvious selective value for inflorescence phyllotaxis suggests that it could in fact be the pleiotropic result of selection on another attribute that has nothing directly to do with floral display or fruit dispersal.

### Genes controlling inflorescence architecture

Many proteins are known to control the architecture of inflorescences in the grasses (Bommert et al., 2005b), and there are good candidate genes for control of the phenotypes described here. The phyllotaxis of primary inflorescence branches and the ultimate fate of the inflorescence meristem are both affected by proteins that regulate meristem size, notably by proteins in the CLAVATA pathway, which together regulate WUSCHEL-like gene expression (Barton, 2010). In grasses, the CLAVATA-like genes include FLORAL ORGAN NUMBER (FON1) in rice [orthologous to THICK TASSEL DWARF (TD1) in maize] and FASCIATED EAR2 (FEA2) in maize (Suzaki et al., 2004, 2006; Bommert et al., 2005a; Chu et al., 2006; Moon et al., 2006). The WUSCHEL-like genes are less well-studied and no mutants are available, but their basic developmental function is inferred to be similar to those in *Arabidopsis*. Mutations in FON-like genes lead to greatly enlarged inflorescence meristems; while they affect phyllotaxis they also disrupt many aspects of normal inflorescence patterning. ABPHYL1, a two-component response regulator, also regulates meristem size (Jackson and Hake, 1999; Giulini et al., 2004). Cytokinin induces transcription of *abphyl1*, which appears then to limit the size of the shoot meristem.

**Figure 12:**
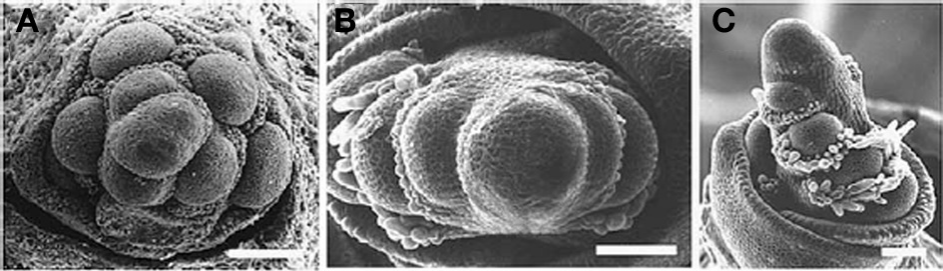
**Early development of the inflorescence of *Oryza sativa*. (A)** wild type. **(B,C)**, *apo1* mutant. Reproduced with permission from Ikeda et al. (2005).

Other proteins affect phyllotaxis by changing the timing of steps in inflorescence development. For example, TERMINAL EAR1 (TE1) of maize also affects phyllotaxis by shortening plastochron length so that the meristem produces lateral organs in a spiral (Veit et al., 1998). Mutations in *ABERRANT PANICLE ORGANIZATION1 APO1*) of rice convert the normally spiral phyllotaxis of the inflorescence to two-ranked (Ikeda et al., 2005, 2007; Ikeda-Kawakatsu et al., 2009), apparently by hastening the conversion of branch meristems to spikelets. APO1 is an F-box protein and is homologous to UNUSUAL FLORAL ORGANS1 (UFO1) of *Arabidopsis*, FIMBRIATA of *Antirrhinum*, DOUBLETOP of *Petunia*, PROLIFERATING FLORAL ORGANS of *Lotus*, and STAMINA PISTILLOIDA of pea, all of which affect inflorescence architecture (Taylor et al., 2001; Zhang et al., 2003; Souer et al., 2008; Ikeda-Kawakatsu et al., 2009). Mutations in *apo1* in rice create a biased distichous inflorescence much like that seen in *Diarrhena* and Stipeae (Figure 12). The protein APO1 regulates the proliferation of cells in the meristem, and in the process controls when a meristem shifts from branch identity to spikelet identity (Ikeda-Kawakatsu et al., 2009). When APO1 levels are low, as in *apo1* loss of function mutants, the shift to spikelet identity occurs prematurely in inflorescence and branch meristems. Conversely, when APO1 levels are elevated, the shift to spikelet identity is delayed. UFO interacts directly with LFY in *Arabidopsis*, making LFY more effective at activating transcription of downstream flowering genes (Chae et al., 2008). In addition, the interaction of LFY and UFO also appears to be important in bract suppression in *Arabidopsis*, which may have a direct or indirect effect on phyllotaxy (Hepworth et al., 2006). However, the action of APO1 in grasses appears to be opposite of that of UFO and its orthologs in dicots, in that APO1 appears to increase cell division and delay formation of floral identity (Ikeda-Kawakatsu et al., 2009).

Despite the highly disruptive effects of knock-out mutations in the genes described above, slight modulations in their expression can change branching patterns without affecting other aspects of meristem function. For example, minor alterations in *Fea2* expression affect the number of branch orthostichies (kernel rows) in the maize ear (Bommert et al., 2013). In addition, *Apo1* has recently been shown to be the gene underlying the QTL *Primary Branch Number* in rice; higher levels of *Apo1* transcript lead to more primary branches and lower levels lead to fewer (Terao et al., 2009).

In summary, we have shown that two-ranked phyllotaxis is apparently synapomorphic for the grass subfamily Pooideae, excluding *Brachyelytrum*, and that the two-ranked phyllotaxis gives rise to distichous phyllotaxis somewhat later in the evolution of the subfamily. Analogous changes occur in other clades as well but appear more sporadic in evolutionary time. We hypothesize that the change in phyllotaxis may be caused by a change in meristem size or aspect ratio. Finally, we hypothesize that modulation of APO1 levels, perhaps relative to LFY levels, could create the observed phenotypic variation, although variation in the CLV pathway could also be involved.

### Conflict of interest statement

The authors declare that the research was conducted in the absence of any commercial or financial relationships that could be construed as a potential conflict of interest.
